# The Impact of Epithelial–Mesenchymal Transition and Metformin on Pancreatic Cancer Chemoresistance: A Pathway towards Individualized Therapy

**DOI:** 10.3390/medicina58040467

**Published:** 2022-03-23

**Authors:** Aiste Gulla, Urte Andriusaityte, Gabrielius Tomas Zdanys, Elena Babonaite, Kestutis Strupas, Helena Kelly

**Affiliations:** 1 Institute of Clinical Medicine, Clinic of Gastroenterology, Surgery, Nephrology, Faculty of Medicine, Vilnius University, Santariskiu Str. 2, 08661 Vilnius, Lithuania; kestutis.strupas@santa.lt; 2 Center of Visceral Medicine and Translational Research, Department of Surgery, Georgetown University Hospital, 3800 Reservoir Road Northwest BLES Building 1st. Floor, Washington, DC 20007, USA; 3 Faculty of Medicine, Vilnius University, M. K. Čiurlionio Str. 21, 03101 Vilnius, Lithuania; urte.andriusaityte@mf.stud.vu.lt (U.A.); zdanys.gabrielius@gmail.com (G.T.Z.); elena.babonaite@mf.stud.vu.lt (E.B.); 4 School of Pharmacy and Biomolecular Sciences, RCSI University of Medicine and Health Sciences, 123 St. Stephen’s Green, D02 YN77 Dublin, Ireland; helenakelly@rcsi.com

**Keywords:** cancer stem cells, chemotherapy, epithelial–mesenchymal transition, metformin, pancreatic ductal carcinoma

## Abstract

Globally, pancreatic ductal adenocarcinoma remains among the most aggressive forms of neoplastic diseases, having a dismal prognostic outcome. Recent findings elucidated that epithelial–mesenchymal transition (EMT) can play an important role in pancreatic tumorigenic processes, as it contributes to the manifestation of malignant proliferative masses, which impede adequate drug delivery. An organized literature search with PubMed, Scopus, Microsoft Academic and the Cochrane library was performed for articles published in English from 2011 to 2021 to review and summarize the latest updates and knowledge on the current understanding of EMT and its implications for tumorigenesis and chemoresistance. Furthermore, in the present paper, we investigate the recent findings on metformin as a possible neoadjuvant chemotherapy agent, which affects EMT progression and potentially provides superior oncological outcomes for PDAC patients. Our main conclusions indicate that selectively suppressing EMT in pancreatic cancer cells has a promising therapeutic utility by selectively targeting the chemotherapy-resistant sub-population of cancer stem cells, inhibiting tumor growth via EMT pathways and thereby improving remission in PDAC patients. Moreover, given that TGF-β1-driven EMT generates the migration of tumor-initiating cells by directly linking the acquisition of abnormal cellular motility with the maintenance of tumor initiating potency, the chemoprevention of TGF-β1-induced EMT may have promising clinical applications in the therapeutic management of PDAC outcomes.

## 1. Introduction

Pancreatic cancer is among the most malignant and aggressive forms of neoplastic diseases, with pancreatic adenocarcinoma (PDAC) accounting for 90% of all pancreatic tumors [1,2]. Although the 5-year survival rate has increased from 5% to 9% over the past decade, this still represents a dismal prognostic outcome [3,4]. The best chance of survival is for patients to be diagnosed at an early stage of PDAC, thus making them eligible for surgery; however, this represents only 20–30% of patients. Therefore, for many patients, surgery is not an option and current chemotherapy regimens, such as FOLFIRINOX and gemcitabine plus nab-paclitaxel, have limited effectiveness. As a result, there is a need to develop novel therapies, including immunotherapies and stroma-targeted methods [5]. Pancreatic tumors are dense, fibrotic masses that impede adequate drug delivery. The main histological hallmark of pancreatic cancer is the heterogeneous tumor microenvironment that dynamically evolves during neoplastic development due to an accumulation of mutations, which transforms normal mucosal cells to precursor lesions and finally to invasive malignancies [6,7]. Understanding the molecular pathways involved in the carcinogenesis, progression, metastasis, and tumor microenvironment growth are crucial for developing effective new therapies [7]. A particular feature of PDAC that has been identified, which significantly contributes to the dense tumor stroma and invasive potential, is the presence of epithelial–mesenchymal transition (EMT) [5]. Due to the abnormal pathological process of EMT, cancer cells obtain a mesenchymal phenotype and express specific extracellular matrix components, which substantially contributes to their migratory capacity. Moreover, various experiments elucidated that EMT gives rise to a specific pluripotent stem cell subpopulation, called cancer stem cells (CSCs), which have self-renewal properties and the ability to promote tumorigenicity [5]. As is explained further below, EMT also participates in specific cellular pathways related to the inhibition of autophagy and cell apoptosis, potentially causing epigenetic changes and a reduced expression of nucleoside transporters, thereby reducing the uptake of chemotherapeutic drugs. All of the above-mentioned factors affect drug transmission efficacy and greatly impact chemoresistance in PDAC. Consequently, research has increasingly focused on the necessity of a therapy addressing the dense stromal environment of the tumor, of which EMT is a contributing factor. Accumulating evidence revealed the potential of the anti-diabetic drug metformin to reverse EMT through diverse signaling pathways, thereby re-establishing chemosensitivity [8,9]. Metformin, through its ability to prevent the overexpression of EMT markers, such as ZEB, TWIST and SLUG (SNAIL2), is able to suppress the dynamic invasiveness and CSC formation in response to the neoplastic microenvironment. Furthermore, metformin inhibits EMT and PDAC metastatic potential through the downregulation of the mTOR pathway; hence, metformin’s ability to inhibit the EMT trans-differentiation process may represent a promising therapeutic strategy to clinically overcome chemotherapy refractoriness in CSC-enriched invasive pancreatic carcinomas [10].

## 2. Materials and Methods

We performed an organized literature search with PubMed, Scopus, Microsoft Academic and the Cochrane library. Inclusion criteria were articles that were published in English from 2011 to 2021. The quality of the articles was judged upon whether information contained within had any significant relevance to EMT in pancreatic cancer and its relationship to chemoresistance, as well as the novel therapy, metformin, as a possible anti-cancer agent. The following main keywords were used: “Epithelial mesenchymal transition”, “Cancer Stem Cells”, “Pancreatic ductal carcinoma”, “metformin” and “chemotherapy”.

## 3. Epithelial–Mesenchymal Transition

### 3.1. Epithelial–Mesenchymal Transition and Cancer Stem Cells

**Figure 1:**
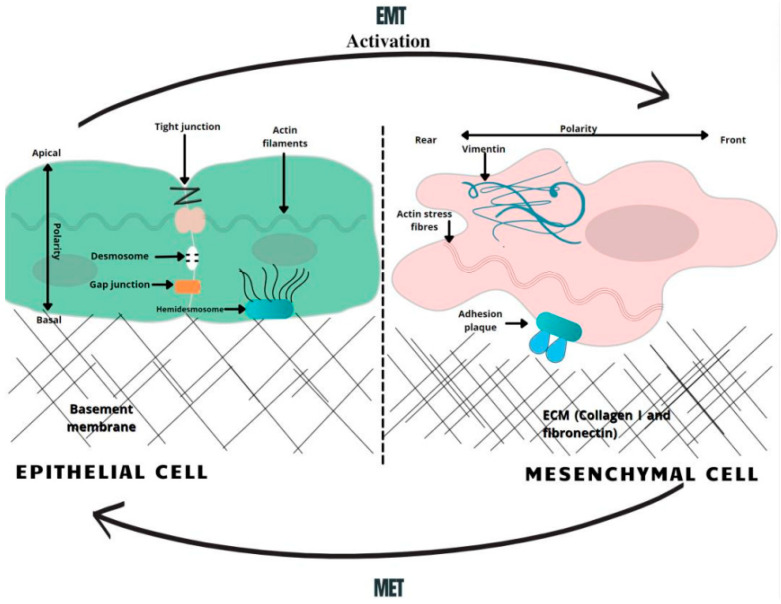
A schematic overview of EMT-related changes in the cell morphology. In the course of EMT, cells lose their normal tight, adherent and gap junctions, retaining only minimal cellular connections. Correspondingly, the adhesion belt made of actin filaments is changed to loose actin stress fibers. Therefore, the activation of the EMT generates profound modulations in cell physiology, especially affecting cell–cell junctions, cytoskeletal arrangement, cell–cell interactions, and the composition of the extracellular matrix (ECM), as well as completely changing cell polarity [26]. MET—mesenchymal-to-epithelial transition, EMT—epithelial-to-mesenchymal transition.

Epithelial cells in solid tumors are observed to undergo EMT, an abnormal pathological process in which cancer cells acquire a mesenchymal phenotype in response to signals they attain from the malignant microenvironment, particularly from reactive stromal cells [11]. Likewise, cells obtain migratory capacity, resistance to apoptosis and expression of extracellular matrix components [12]. These changes generate CSCs, a subset of undifferentiated neoplastic cells that can initiate heterogeneous cancers while triggering tumorigenesis. According to the “Seed and Soil” hypothesis, first proposed by Stephen Paget in 1889, cancer-initiating cells are endowed with higher tumor-forming potential and have the capacity to “reproduce” neoplasms when cells seed distant organs to develop metastases [13]. In PDAC, cancer initiating cells have been specified as those expressing CD44, CD24 and ESA, forming tumors at a higher frequency, compared to cells that do not express this phenotype [14]. In recent studies, it was demonstrated that more than 65% of PDAC patient-derived organoids had characteristics of CSC, characterized as CD44+ CD24+ [15]. The EMT phenomenon has been directly related to cancer stem cell features, since CSCs also express an EMT phenotype in various types of cancer, including PDAC. In an analysis of breast cancer, it was observed that the upregulation of EMT transcriptional factors, such as TWIST or SNAI, made the cells more mesenchymal and elevated the expression of CD44 and CD24 [15]. It was also proposed that the EMT regulator ZEB1 enforces isoforms of CD44, while CD44s activates the expression of ZEB1. As a result, a self-sustaining expression of ZEB1 and CD44 is achieved, thereby linking EMT with the stem cell properties in PDAC [16]. Therefore, it can be considered that EMT contributes to pancreatic cancer stem cells’ migratory capacity by maintaining their ability to multiply and participate in the production of progenies in metastasis. Apart from CD44+ CD24+ ESA+ populations of pancreatic cancer stem cells, c-Met (mesenchymal–epithelial transition factor) has also been identified as a potential prognostic factor for PDAC [17]. c-Met is a receptor tyrosine kinase found on the surface of epithelial cells. In normal circumstances, c-Met and its ligand HGF/SF (hepatocyte growth factor/scatter factor) moderate tissue regeneration, wound healing and the formation of nerves and muscles [18]. The abnormal activation of c-Met can promote the development and progression of multiple cancers [19]. For instance, a recent analysis compared the median survival for PDAC patients, revealing 21.65 months of viability for negative c-Met patients and only 9.45 months for positive ones [20]. In this regard, high c-Met expression is closely associated with poor prognosis in PDAC patients. Several other processes that occur during EMT include activation of transcription factors, such as the key regulators, SNAI1, SNAI2, ZEB, and TWIST factors [21]. These EMT regulators have been shown to repress the expression of E-cadherin, a key epithelial marker. The downregulation of E-cadherin impedes cellular adhesion and imparts cellular motility, which is correlated with reduced chemotherapeutic drug sensitivity [22,23]. Concurrently, the activation of snail proteins upregulates the expression of mesenchymal proteins, such as vimentin [24]. Vimentin is usually found as the main intermediate filament protein of normal mesenchymal tissue. Vimentin functions to maintain cellular integrity and provides resistance against stress factors, and its expression has been detected in epithelial malignancies, such as gastrointestinal tumors. During EMT, pancreatic cells change expression from keratin- to vimentin-type intermediate filaments and become resistant to programed cell death [25] (Figure 1).

### 3.2. Chemoresistance and EMT Impact on Oncotherapy

Taking into consideration all of the above-mentioned mechanisms, it is now widely accepted that the EMT-associated modifications of gene expression and CSC populations both have crucial roles in making cells resistant to current clinical therapies [26,27]. Dynamic and proliferative CSC populations with high cellular plasticity, including a unique self-renewal capacity, which is critical for metastasis and chemoresistance, subsequently makes them resistant to conventional chemotherapy; hence, CSCs are a crucial target when considering novel treatments [28]. Moreover, traditional chemotherapy targets proliferating cells, while CSCs have been widely described as being slow-cycling and quiescent, which is a prominent mechanism for resistance to the conventional therapies [29]. Hence, CSC-targeted chemotherapeutic regimens have the potential to suppress tumor progression, inhibit their metastasis-forming ability and ultimately overcome therapeutic resistance, including relapses, which are frequently observed in response to current treatment methods [30]. Studies with gemcitabine, 5-FU (5-fluorouracil) and cisplatin have shown that EMT-type cells are resistant to chemotherapy, while non-EMT-type cells are susceptible to these chemotherapeutic drugs. Altogether, EMT plays an important role in the regulation of treatment resistance in PDAC, suggesting that targeting EMT could not only reduce and suppress the population of CSCs that have been implicated in tumor metastasis, but also contribute to an increased sensitivity to chemotherapy [31].

Collectively, EMT is involved in mediating NF-kB and AKT activity, elements that are known to be major regulators of autophagy processes, with both leading to cell apoptosis [23]. EMT influences neoplastic cells to gain a CSC phenotype and enhances their neoplastic replicative capacity, as well as increasing chemotherapeutic resistance [32,33]. Other factors determining EMT-initiated chemoresistance include epigenetic changes, which reduce the expression of the hENT1 nucleoside transporters needed for gemcitabine uptake. CSCs showing an EMT phenotype are prone to express ATP-binding cassette (ABC) transporters [34]. The overexpression of ABC family efflux transporters also increases the efflux of therapeutic agents from cells, e.g., the MDR1 and MRP1 ABC efflux transporters initiate a resistance to nucleoside analogues, such as gemcitabine [35]. EMT also initiates the activation of inert fibroblasts in the tumor microenvironment by modifying epithelial cells to active myofibroblasts. Fibro-inflammatory stroma (the desmoplastic stroma) is made of dense ECM deposited mostly by cancer-associated fibroblasts [36,37]. A dense stroma surrounds the tumor and increases the tumor interstitial fluid pressure, leading to poor tumor elasticity and the decreased perfusion and efficacy of administered therapy [33].

Furthermore, EMT activates specific mechanisms that help cancer cells avoid the effect of cytotoxic T cells. These include increased expression of PD-L1, which binds to inhibitory immune-checkpoint receptors, expressed by T cells, thereby diminishing their function. The activity of T cells is also inhibited by the elevated secretion of thrombospondin-1, which normally stimulates the maturation of T cells in the tumor microenvironment. The inhibition of thrombospondin-1 consequently disrupts the activity and development of T cells [26].

**Figure 2:**
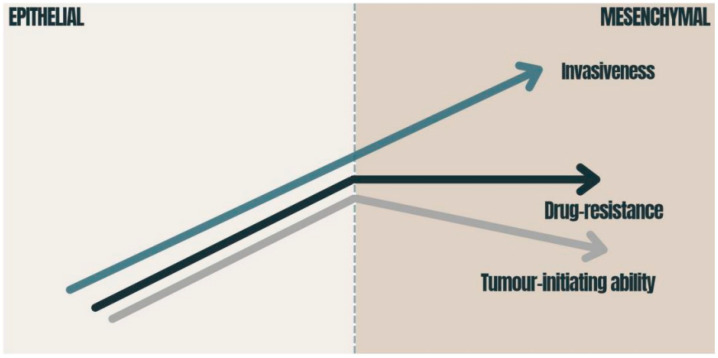
Consequences of EMT in carcinomas: the graphic presents how the tumor-initiating ability, invasiveness and degree of chemoresistance change during EMT activation. The tumor-initiating ability is affected by the degree of EMT activation; immense EMT activation has a deleterious effect on the tumor-initiating ability [40]. Drug resistance is also related to EMT and reaches its maximum at an intermediate level of EMT activation [40,41]. The migration of cancer cells requires the strong activation of the EMT program [42]. EMT—epithelial-to-mesenchymal transition.

EMT therefore plays a critical role in inducing stem cell features, which have been linked to enhanced invasiveness and aggressiveness, ensuring that cell dissemination from the primary tumor location to the metastasis site occurs even before pancreatic cancer lesions are detected [38]. This phenomenon helps to explain the parallel low survival rate of PDAC patients with increased EMT, whereby they have an impaired capacity to respond to current chemotherapeutic methods [39] (Figure 2).

## 4. Metformin and PDAC

Metformin is the most widely used oral anti-hyperglycemic agent in the treatment of type 2 diabetes, where it acts to lower glucose through enhancing the activity of insulin [43,44]. However, a number of epidemiological studies identified an association of improved survival outcomes among PDAC patients being treated with metformin in combination therapy (compared to those receiving a monotherapy) [45,46]. With a random model approach, there are important differences in the overall survival (HR = 0.85, 95% CI: 0.77–0.94, *p* = 0.002) between PDAC patients who were treated with metformin and underwent pancreatectomy and those who underwent pancreatectomy without metformin treatment [47]. A novel retrospective study of diabetic patients with pancreatic cancer also found that the mean overall survival time was 15.2 months for the metformin-treated group and 11.1 months for the control group. These results were only statistically significant in patients with non-metastatic lesions [48]. Thus, the correlation between metformin usage and its role in beneficial outcomes on PDAC treatment is now a focus of ongoing research to identify effective novel therapeutic strategies for PDAC.

Metformin has been identified as having the potential to modulate multiple molecular pathways crucial to malignant cell progression and metabolism by inhibiting features of aggressive tumors, such as desmoplasia and EMT reactions. The desmoplastic reaction, resulting from the proliferation of activated pancreatic stellate cells (PSCs) and the increased deposition of extracellular matrix components, is a significant pathological characteristic of pancreatic neoplasms, while EMT is responsible for invasion-metastasis cascades in pancreatic cancer cells [49]. As TGF-β1 is a potent inducer of EMT, accumulating evidence suggests that an inhibition of TGF-β1 production in PDAC cells by metformin-induced AMPK activation inhibits malignant pancreatic cell growth [50].

The novel advances elucidating the molecular mechanisms that are relevant for metformin usage in PDAC treatment demonstrated that it can induce the apoptosis by augmenting the activity caspases, such as caspase-3 and -8. Caspase-8, as a highly promising putative target, leads to the activation of caspase-3, -6, and -7, which directly induce the cell-death pathway [51]. Furthermore, metformin inhibits neoplastic angiogenesis via suppressing the HIF-1α-induced expression of angiogenesis-associated factors (AAFs) and downregulating the expression of the vascular endothelial growth factor (VEGF) [52]. By the same token, metformin demonstrates a great promise in improving current cancer therapies, as it inhibits the matrix metalloproteinases ((MMP)-2 and (MMP)-9) and suppresses the migration of tumorigenic endothelial cells. One of the most important factors in the metastasis of PDAC cells is matrix metalloproteinase MMPs, which destroys the extracellular matrix and allows malignant pancreatic cells to form metastasis. These anticancer properties appear to synergize with already existing chemotherapeutics, which allow us to postulate that metformin might play a role in PDAC prevention and therapeutic treatment, whereas it counteracts malignant cell development and growth on specific levels [51,53].

### 4.1. Role of mTOR Mediation and CSC in an AMPK-Dependent Manner

One of the leading ways in which metformin exerts its anti-tumor effect is via the AMP-activated kinase signaling network, where it suppresses malignancy through the inhibition of cellular proliferation and division via the suppression of the downstream mammalian target of rapamycin (mTOR)-signaling pathways and the AMP-activated protein kinase axis, which induces apoptosis and autophagy of cancer cells [54]. Collectively, these observations indicate that TGF-β1-driven EMT is responsible for the migration of tumor initiating cells by directly linking the acquisition of neoplastic cellular motility with the maintenance of malignant proliferation potency; thus, metformin-mediated TGF-β1 downregulation may be responsible for suppressed migration, proliferation, abnormal malignant cell invasion and EMT changes [55,56].

Hence, metformin could induce signal transduction inhibition and potentiate the effects of various chemotherapeutic drugs (e.g., 5-fluorouracil, gemcitabine, and cisplatin), which are current treatment options for PDAC [8]. In addition, the suppression of cytokine production by PDAC cells may be a fundamental underlying cause of these metformin-mediated effects, as cancer cell-derived cytokines are crucial elements in inducing PSC activation, which are the major cellular components of the desmoplastic reaction in PDAC [23,57].

In addition, pancreatic CSCs can be inhibited by metformin directly through the mTOR pathway, in which it blocks tumor growth by suppressing neoplastic proliferation, resulting in malignant cell apoptosis [58]. Given the evidence of beneficial anti-tumor effects as well as its low toxicity and low cost, compared to other antineoplastic drugs, metformin has the potential to become a promising anticancer agent due to its tolerability, widespread availability, oral administration and its potential for chemosensitization. Autophagy, an evolutionarily conserved process allowing cells to recycle and eliminate damaged or unused cytoplasmic constituents and cellular components, has been shown to be upregulated in PDAC [59]. In abnormal malignant cells, autophagy maintains neoplastic progression and anticancer therapy resistance, by providing a means for cells to survive intracellular and extracellular stress [60]. Consequently, autophagy inhibition is regarded as a potential therapeutic target in PDAC and considering mTOR as a fundamental regulator of the autophagy pathway, the strategy of directly targeting mTOR with metformin has been considered as a novel potential central aim in anti-neoplastic and chemopreventive therapy models [61,62]. Cumulative evidence suggests that pancreatic cancer cells treated with metformin have significantly increased sensitivity to chemotherapy [61,63]. This enhanced sensitivity was due to metformin’s potential antineoplastic efficacy mediated through molecular mechanisms of the mTOR signaling pathway and the AMP-activated protein kinase axis. The beneficial anti-tumor outcome is induced through AMPK signaling, which suppresses mTOR activity. This inhibition of mTOR results in reduced protein synthesis and translation both with suppressed abnormal cancer cell cycle progression and proliferation [64,65].

By the same token, metformin regulates PDAC carcinogenesis not only through mTOR activation but also via AMPK-mediated and AMPK-independent mechanisms by up-regulating the expression of REDD1 to cause cancer cell cycle arrest [66]. REDD1 itself is a negative regulator of the mTOR pathway, and metformin enhances REDD1 expression in a p53-dependent manner [67]. Therefore, suppression of mTOR in specific KRAS-dependent PDAC subtypes leads to the impediment of tumorigenesis and furthermore, over 90% of PDAC patients have oncogenic KRAS mutations; thus, suppressing the p53/REDD1 axis as a molecular central target, may increase metformin’s potential anti-tumor effect to become a novel promising chemotherapeutic agent in the foreseeable future [68,69].

### 4.2. Metformin and miRNAs

MicroRNAs (miRNAs) have an important role in the regulation of cell growth and proliferation, differentiation, apoptosis and energy metabolism. At a molecular level, miRNAs downregulate gene expression, contributing to mRNA degradation or inhibition of translation [70]. Through these activities miRNAs impact the phenotypes of cancer initiating cells and the progression of tumors, where deregulated miRNAs might act as either oncogenes or tumor suppressor genes [71]. Increasing evidence shows that miRNAs have a role in the malignant behavior of cancer cells, such as EMT-related cancer metastasis [72]. Recent studies demonstrated that miRNAs act by inhibiting specific genes related to EMT and might be critically involved in regulating drug resistance in pancreatic cancer [73,74]. Using the scratch wound healing assay, it was observed that a specific miRNA, named miR-125a-3p, decreased the migratory capacity of PDAC cells with greatly inhibited cell invasion. In addition, the expression of mesenchymal markers, N-cadherin, Slug, Vimentin and Snail, were downregulated, while epithelial markers, such as E-cadherin, were upregulated in miR-125a-3p transfected PDAC cells [75].

Further research (Hiramoto et al. [76]; Funamizu et al. [71]) illustrated that miR-200b, miR-509-5p or miR-1243 overexpression could also increase sensitivity to gemcitabine by suppressing EMT-related gene expression and upregulating E-cadherin expression in PDAC. It was also shown that after transfecting Panc1 cells with any of these miRNAs, the protein expression of ZO1 (a tight junction protein) was increased, while the expression of representative EMT marker genes, ZEB1 and Snail, was reduced [76,77].

Metformin has also been shown to target some miRNAs and specific genes in CSCs, which, in turn, inhibits the mRNA levels of EMT-specific markers, such as ZEB1, TWIST1, and SNAIL2 transcription factors [67]. Since metformin upregulates specific miRNA expression (such as miR-141, miR-205, and miR-429), the reversal of EMT might be simultaneously induced [78]. It was observed that in pancreatospheres originating from gemcitabine-resistant pancreatic cancer cells, metformin upregulated specific miRNAs, which are normally downregulated in PC cells. The re-expression of these miRNAs contributed to lower pancreatosphere formation and a reduction in mRNA levels of CSC markers [79].

Another convincing novel study also compared the effect of metformin on miRNA expression in cells cultured in different glucose concentrations. There was a more pronounced inhibitory effect of metformin on PANC-1 cells in low glucose conditions compared to a high glucose state, which was related to the upregulated expression of miR-210-5p, which concurrently inhibited glycolysis via the enzyme PFKFB2. Based on these promising results, there is speculation that increased levels of miR-210-5p might enhance the anti-tumor effects on cells under low glucose conditions [80].

### 4.3. CSCs’ Resistance to Metabolic Deprivation: A Potential Target in PDAC Treatment?

Based on the tenets of the “Seed and Soil” theory, once metastatic tumor cells extravasate into a foreign tissue, their ability to successfully colonize and proliferate depends on their capacity to respond to their new microenvironment [81]. Owing to this theory, in which Virchow and Paget considered the evolution of a specific malignant cell in a main primary tumor, we gained an understanding of how cells obtain a particular metabolic signature that sustains their prospective metastatic invasion and colonization pathways in a certain organ or organ system [13,81]. Aggressive solid tumors are enriched by pluripotent embryonic cancer stem cell genes; thus, a relevant mutual relationship exists among the neoplasm microenvironment and tumor-initiating cells [82].

Several recent observational studies explored the parallels among specific cancer cell subpopulations, their proliferation capacity, metabolism and organ-specific colonization with a metastasis-forming ability [13]. CSCs within tumors may haphazardly disseminate, and only those neoplastic units that metabolically match the distant site will colonize, disperse and develop a metastasis in a short-term perspective [81]. These specific CSC potencies determine tumor origin and tumor cell heterogeneity, playing a key role in PDAC tumorigenesis, drug resistance, invasive development and the abnormal metabolism of neoplastic cells along with the capability of metastasis [83].

Other studies elucidated that pancreatic tumor-initiating cells can exhibit a highly mitochondrial-dependent metabolic profile as well as immense tolerance to nutrition deprivation, which enables them to endure a hypovascular tumor microenvironment. This is a striking difference compared to regular stem cells without any abnormal neoplastic deviations, but it also distinguishes them from the bulk of pancreatic cancer cells. Thus, anti-diabetic medicaments, such as metformin, which targets oxidative mitochondrial metabolism, can exert powerful chemotherapeutic potency in order to induce a fatal energy crisis and eliminate CSCs [67,84]. In addition, metformin enhances reactive oxygen species (ROS) production in tumor cells and reduces mitochondrial transmembrane potential, thereby selectively eradicating pancreatic CSCs in stark contrast to the chemotherapeutical effects of gemcitabine, a medicament that annihilates only bulk tumor cells, but not the cancer initiating cells [85].

Targeting CSCs through the inhibition of mitochondrial function is a novel chemotherapeutic development in PDAC, whereby CSCs dependent on the mitochondrial oxidative phosphorylation system (OXPHOS) can be eliminated by impairing mitochondrial energy metabolism. Furthermore, OXPHOS signatures of PDAC could be significant in patient selection as most malignant cells use OXPHOS as major source of metabolism. Thus, this represents a promising up-and-coming treatment niche as the direct inhibition of OXPHOS can be achieved with antidiabetic agents, such as metformin, which cause tumor cell death by evoking an energy crisis in CSCs [86]. However, the most promising and effective treatment results were attained in pre-clinical studies with a combination of gemcitabine and metformin, indicating that this combined chemotherapy regimen potently restricts the growth of gemcitabine-resistant PDAC through suppressing the function of mTOR in malignant cells [87].

### 4.4. Clinical Trials

To date, there have been very few clinical trials to evaluate the hypothesis of metformin as an adjunct treatment modality in PDAC. At the time of writing there are, according to clinicaltrials.gov, 21 clinical trials testing the pharmacological outcomes of metformin on PDAC. Only nine of them have been completed, however, the results are not available to the public, while six are recruiting, four have been terminated, and two are active. Currently the best knowledge comes from a study in the Netherlands, where 121 patients with inoperable PDAC were randomized to receive either gemcitabine/erlotinib plus 2 × 500 mg metformin daily or gemcitabine/erlotinib and placebo. There was no statistically significant difference in overall survival at 6 months (median 7.6 vs. 6.8 months, log-rank test *p* = 0.78, HR 1.06, 95% CI 0.72–1.55) [85]. However, it was noted that the metformin group had a significantly higher CA19-9 level compared to the placebo group: 561 and 245 kU/L, respectively. Although CA19-9 is not particularly sensitive marker, this raises the possibility that the metformin group had proportionately more advanced cancers to begin with, creating some questions regarding validity of the study. This difference could have resulted in poorer absorption and/or tolerance of metformin and, thus, the desired therapeutic effect might not have been attained [88,89]. In another randomized phase II trial, involving 60 patients receiving cisplatin, epirubicin, capecitabine, and gemcitabine with (n = 31) or without (n = 29) metformin, 6 months progression-free survival was 52% (95% CI, 33–69) in the control group and 42% (95% CI, 24–59) in the metformin group (*p* = 0.61). Furthermore, there was no difference in the disease-free survival and overall survival between the groups [90].

In a pre-clinical trial of a murine orthotopic PDAC tumor model, a combination of bis-2-(5-phenylacetamido-1,2,4-thiadiazol-2-yl) ethyl sulphide 3 (BPTES) and metformin was evaluated. The combination with metformin was investigated as metformin inhibits respiratory complex 1, which catalyzes the transfer of electrons from NADH to coenzyme Q10, thereby decreasing the synthesis of ATP. This increases the ratios of AMP/ATP and ADP/ATP, leading to the activation of AMPK. Consequently, AMPK inhibits mTOR, which regulates numerous cellular pathways, including translation [89,91]. Therefore, the combination of BPTES and metformin has the potential to target multiple metabolic pathways and confer anticancer effects. During this pre-clinical trial, in which the team injected 54 mg/kg BPTES and 250 mg/kg metformin via intraperitoneal injection, it was concluded that a combination of metformin and BPTES resulted in a greater tumor growth inhibition compared to the monotherapy alone. The authors also emphasized the possibility that the combination therapy with metformin could be effective against PDAC because metformin targets slowly dividing hypoxic cells, which are reliant on glucose [92].

However, on the other hand, while some cohort studies support a favorable role of metformin in PDAC patients, data from randomized controlled trials (RCTs) have failed to corroborate these findings. Pooled RR from 6 RCT studies showed no relationship between metformin and mortality in PDAC patients (RR 0.93, 95% CI 0.82, 1.05; *p* = 0.22 and I2 = 75%) [93]. An observational cohort study (of PDAC patients?) analyzed 77 users of metformin, 43 users of sulfonylurea derivatives, and 787 non-users. The adjusted rate ratio for overall survival for metformin users vs. non-users was 0.86 (95% CI: 0.66–1.11; *p* = 0.25). Therefore, in the end, no association was detected between overall survival PDAC and metformin use [94]. The meta-analysis of 2 RCTs with 181 pancreatic cancer patients showed that metformin use was not associated with improved survival at 6 months (RR = 0.90, 95% CI = 0.67–1.21), overall survival (HR = 1.19, 95% CI = 0.86–1.63) or progression-free survival (HR = 1.39, 95% CI = 0.97–1.99) [95]. To conclude, further prospective studies are required in order to understand these associations (and the potential positive benefits of metformin on PDAC outcome) better.

## 5. Conclusions

Epithelial–mesenchymal transition (EMT) initiates an invasion-metastasis cascade, which is a feature of aggressive tumors and plays a crucial role in the invasion, metastasis and drug resistance of PDAC. EMT is related to modulations of NF-kB and AKT activity, the main regulators of autophagy processes as well as cell apoptosis. Additionally, the presence of EMT in the solid tumor microenvironment influences neoplastic cells to attain the CSC phenotype, thereby enhancing their neoplastic replicative capacity.

Novel clinical studies demonstrated that metformin enhances the antiproliferative effects of pancreatic cancer cells, inhibiting EMT and potentially improving an ultimate prognosis of PDAC patients. Its promising safety properties and widespread access are the main keys to its potential feasibility. Therapeutic metformin exposure is associated with decreasing morbidity and mortality rates, along with modestly augmenting short-term survival rates and unaffecting the long-term survival rates of PDAC patients. In connection with epidemiological and pre-clinical research, data support the fact that metformin requires strict prospective clinical trials to identify those who might obtain advantage from metformin combinations with other chemotherapeutic agents. Therefore, metformin usage might be associated with better survival benefit for pancreatic cancer patients, depending on individual pathological features, pre-operative physiology, operative results and further adjuvant chemotherapy. Yet, given that the survival outcomes are affected by multiple clinical factors, such as the type of malignancy, its differentiation, staging and individual treatment regime, for adequately repurposing the use of metformin in PDAC, it is essential to take into consideration the specific tumor-driven oncogenic pathway, as well as individual clinical patient characteristics.

## 6. Future Directions

Considering that current clinical evidence related to metformin is associated with only pre-clinical data and that pancreatic cancer cells are sensitive to the antineoplastic effects of metformin, no concrete conclusion can be drawn, particularly due to the retrospective non-randomized nature of these studies. Hence, there is a need of future clinical trials that would incorporate the identification of the disease stage, biomarkers and chemotherapeutic treatment setting to assess the advantage of metformin in the treatment of PDAC. Thus, even supposing that modulation of metabolism and cell cycle arrest are potential therapeutic cancer targets utilized by metformin to boost the anti-cancer effects of chemotherapy, there is a need of studying metformin effects in more specific clinical scenarios and confirm its benefit prospectively in comprehensive future clinical trials.

Nevertheless, to date, a paramount number of new alternative therapeutic approaches, such as DNA repair strategies, immunotherapy vaccines or RNA interference, emerge. There are successful preclinical models that target specific somatic driver mutations and demonstrate the potential inhibition of pancreatic tumor growth. Simultaneously, vitamin D analogues are being analyzed since they inhibit EMT deposition in a preclinical set up. Together with new preclinical strategies, there are new recommendations to start molecular subtyping and routine germline testing in all PDAC patients. All these important developments lead to targeted, individual therapies and encourage optimism related to improved survival and outcomes in this disease [96].

## Data Availability

Not applicable.

## References

[1] Vincent A., Herman J., Schulick R., Hruban R.H., Goggins M. (2011). Pancreatic cancer. Lancet.

[2] Rawla P., Sunkara T., Gaduputi V. (2019). Epidemiology of Pancreatic Cancer: Global Trends, Etiology and Risk Factors. World J. Oncol..

[3] Bray F., Ferlay J., Soerjomataram I., Siegel R.L., Torre L.A., Jemal A. (2018). Global cancer statistics 2018: GLOBOCAN estimates of incidence and mortality worldwide for 36 cancers in 185 countries. CA Cancer J. Clin..

[4] Rahib L., Smith B.D., Aizenberg R., Rosenzweig A.B., Fleshman J.M., Matrisian L.M. (2014). Projecting cancer incidence and deaths to 2030: The unexpected burden of thyroid, liver, and pancreas cancers in the united states. Cancer Res..

[5] Chandana S.R., Babiker H.M., Mahadevan D. (2019). Therapeutic trends in pancreatic ductal adenocarcinoma (PDAC). Expert Opin. Investig. Drugs.

[6] Hanahan D., Weinberg R.A. (2011). Hallmarks of cancer: The next generation. Cell.

[7] Hessmann E., Buchholz S.M., Demir I.E., Singh S.K., Gress T.M., Ellenrieder V., Neesse A. (2020). Microenvironmental determinants of pancreatic cancer. Physiol. Rev..

[8] Candido S., Abrams S.L., Steelman L., Lertpiriyapong K., Martelli A.M., Cocco L., Ratti S., Follo M.Y., Murata R.M., Rosalen P.L. (2018). Metformin influences drug sensitivity in pancreatic cancer cells. Adv. Biol. Regul..

[9] Chen K., Qian W., Jiang Z., Cheng L., Li J., Sun L., Zhou C.C., Gao L.P., Lei M., Yan B. (2017). Metformin suppresses cancer initiation and progression in genetic mouse models of pancreatic cancer. Mol. Cancer.

[10] Del Barco S., Vazquez-Martin A., Cufí S., Oliveras-Ferraros C., Bosch-Barrera J., Joven J., Martin-Castillo B., Menendez J.A. (2011). Metformin: Multi-faceted protection against cancer. Oncotarget.

[11] Zhou P.T., Li B., Liu F.R., Zhang M.C., Wang Q., Liu Y.H., Yao Y., Li D. (2017). The epithelial to mesenchymal transition (EMT) and cancer stem cells: Implication for treatment resistance in pancreatic cancer. Mol. Cancer.

[12] Beuran M., Negoi I., Paun S., Ion A.D., Bleotu C., Negoi R.I., Hostiuc S. (2015). The epithelial to mesenchymal transition in pancreatic cancer: A systematic review. Pancreatology.

[13] Lehúede C., Dupuy F., Rabinovitch R., Jones R.G., Siegel P.M. (2016). Metabolic plasticity as a determinant of tumor growth and metastasis. Cancer Res..

[14] Gzil A., Zarębska I., Bursiewicz W., Antosik P., Grzanka D., Szylberg Ł. (2019). Markers of pancreatic cancer stem cells and their clinical and therapeutic implications. Mol. Biol. Rep..

[15] Choi J.-I., Jang S.I., Hong J., Kim C.H., Kwon S.S., Park J.S., Lim J.-B. (2021). Cancer-initiating cells in human pancreatic cancer organoids are maintained by interactions with endothelial cells. Cancer Lett..

[16] Preca B.-T., Bajdak K., Mock K., Sundararajan V., Pfannstiel J., Maurer J., Wellner U., Hopt U.T., Brummer T., Brabletz S. (2015). A self-enforcing CD44s/ZEB1 feedback loop maintains EMT and stemness properties in cancer cells. Int. J. Cancer.

[17] Li C., Wu J., Hynes M., Dosch J., Sarkar B., Welling T.H., di Magliano M.P., Simeone D.M. (2011). c-Met is a marker of pancreatic cancer stem cells and therapeutic target. Gastroenterology.

[18] Zhang Y.Z., Xia M.F., Jin K., Wang S.F., Wei H., Fan C.M., Wu Y.F., Li X.L., Li X.Y., Li G.Y. (2018). Function of the c-Met receptor tyrosine kinase in carcinogenesis and associated therapeutic opportunities. Mol. Cancer.

[19] Neuzillet C., Couvelard A., Tijeras-Raballand A., de Mestier L., de Gramont A., Bédossa P., Paradis V., Sauvanet A., Bachet J.-B., Ruszniewski P. (2015). High c-Met expression in stage I–II pancreatic adenocarcinoma: Proposal for an immunostaining scoring method and correlation with poor prognosis. Histopathology.

[20] Lux A., Kahlert C., Grützmann R., Pilarsky C. (2019). c-Met and PD-l1 on circulating exosomes as diagnostic and prognostic markers for pancreatic cancer. Int. J. Mol. Sci..

[21] Jiang J.H., Liu C., Cheng H., Lu Y., Qin Y., Xu Y.F., Xu J., Long J., Liu L., Ni Q.X. (2015). Epithelial-mesenchymal transition in pancreatic cancer: Is it a clinically significant factor?. Biochim. Biophys. Acta-Rev. Cancer.

[22] Lamouille S., Xu J., Derynck R. (2014). Molecular mechanisms of epithelial-mesenchymal transition. Nat. Rev. Mol. Cell Biol..

[23] Schober M., Jesenofsky R., Faissner R., Weidenauer C., Hagmann W., Michl P., Heuchel R.L., Haas S.L., Löhr J.-M. (2014). Desmoplasia and chemoresistance in pancreatic cancer. Cancers.

[24] Gradiz R., Silva H.C., Carvalho L., Botelho M.F., Mota-Pinto A. (2016). MIA PaCa-2 and PANC-1—pancreas ductal adenocarcinoma cell lines with neuroendocrine differentiation and somatostatin receptors. Sci. Rep..

[25] Wu S., Du Y., Beckford J., Alachkar H. (2018). Upregulation of the EMT marker vimentin is associated with poor clinical outcome in acute myeloid leukemia. J. Transl. Med..

[26] Shibue T., Weinberg R.A. (2017). EMT, CSCs, and drug resistance: The mechanistic link and clinical implications. Nat. Rev. Clin. Oncol..

[27] Holohan C., Van Schaeybroeck S., Longley D.B., Johnston P.G. (2013). Cancer drug resistance: An evolving paradigm. Nat. Rev. Cancer.

[28] Sancho P., Alcala S., Usachov V., Hermann P.C., Sainz B. (2016). The ever-changing landscape of pancreatic cancer stem cells. Pancreatology.

[29] Lyle S., Moore N. (2010). Quiescent, slow-cycling stem cell populations in cancer: A review of the evidence and discussion of significance. J. Oncol..

[30] Bhagwandin V.J., Bishop J.M., Wright W.E., Shay J.W. (2016). The Metastatic Potential and Chemoresistance of Human Pancreatic Cancer Stem Cells. PLoS ONE.

[31] Hu X., Chen W. (2021). Role of epithelial-mesenchymal transition in chemoresistance in pancreatic ductal adenocarcinoma. World J. Clin. Cases.

[32] Celià-Terrassa T., Jolly M.K. (2020). Cancer stem cells and epithelial-to-mesenchymal transition in cancer metastasis. Cold Spring Harb. Perspect. Med..

[33] Cannon A., Thompson C., Hall B.R., Jain M., Kumar S., Batra S.K. (2018). Desmoplasia in pancreatic ductal adenocarcinoma: Insight into pathological function and therapeutic potential. Genes Cancer.

[34] Li Y., Kong D., Ahmad A., Bao B., Sarkar F.H. (2013). Pancreatic cancer stem cells: Emerging target for designing novel therapy. Cancer Lett..

[35] Adamska A., Falasca M. (2018). ATP-binding cassette transporters in progression and clinical outcome of pancreatic cancer: What is the way forward?. World J. Gastroenterol..

[36] Awaji M., Singh R.K. (2019). Cancer-associated fibroblasts’ functional heterogeneity in pancreatic ductal adenocarcinoma. Cancers.

[37] Ohlund D., Handly-Santana A., Biffi G., Elyada E., Almeida A.S., Ponz-Sarvise M., Corbo V., Oni T.E., Hearn S.A., Lee E.J. (2017). Distinct populations of inflammatory fibroblasts and myofibroblasts in pancreatic cancer. J. Exp. Med..

[38] Rhim A.D., Mirek E.T., Aiello N.M., Maitra A., Bailey J.M., McAllister F., Reichert M., Beatty G.L., Rustgi A.K., Vonderheide R.H. (2012). EMT and dissemination precede pancreatic tumor formation. Cell.

[39] Aiello N.M., Maddipati R., Norgard R.J., Balli D., Li J., Yuan S., Yamazoe T., Black T., Sahmoud A., Furth E.E. (2018). EMT Subtype Influences Epithelial Plasticity and Mode of Cell Migration. Dev. Cell.

[40] Bierie B., Pierce S.E., Kroeger C., Stover D.G., Pattabiraman D.R., Thiru P., Donaher J.L., Reinhardt F., Chaffer C.L., Keckesova Z. (2017). Integrin-β4 identifies cancer stem cell-enriched populations of partially mesenchymal carcinoma cells. Proc. Natl. Acad. Sci. USA.

[41] Pattabiraman D.R., Bierie B., Kober K.I., Thiru P., Krall J.A., Zill C., Reinhardt F., Tam W.L., Weinberg R.A. (2016). Activation of PKA leads to mesenchymal-to-epithelial transition and loss of tumor-initiating ability. Science.

[42] Clark A.G., Vignjevic D.M. (2015). Modes of cancer cell invasion and the role of the microenvironment. Curr. Opin. Cell Biol..

[43] Schernthaner G., Schernthaner G.H. (2020). The right place for metformin today. Diabetes Res. Clin. Pract..

[44] Gong L., Goswami S., Giacomini K.M., Altman R.B., Klein T.E. (2012). Metformin pathways: Pharmacokinetics and pharmacodynamics. Pharm. Genom..

[45] Lee M.-S., Hsu C.-C., Wahlqvist M.L., Tsai H.-N., Chang Y.-H., Huang Y.-C. (2011). Type 2 diabetes increases and metformin reduces total, colorectal, liver and pancreatic cancer incidences in Taiwanese: A representative population prospective cohort study of 800,000 individuals. BMC Cancer.

[46] Coyle C., Cafferty F.H., Vale C., Langley R.E. (2016). Metformin as an adjuvant treatment for cancer: A systematic review and meta-analysis. Ann. Oncol..

[47] Wan G., Sun X., Li F., Wang X., Li C., Li H., Yu X., Cao F. (2018). Cellular Physiology and Biochemistry Cellular Physiology and Biochemistry Survival Benefit of Metformin Adjuvant Treatment For Pancreatic Cancer Patients: A Systematic Review and Meta-Analysis. Cell Physiol. Biochem..

[48] Sadeghi N., Abbruzzese J.L., Yeung S.C.J., Hassan M., Li D. (2012). Metformin use is associated with better survival of diabetic patients with pancreatic cancer. Clin. Cancer Res..

[49] Duan W.X., Chen K., Jiang Z.D., Chen X., Sun L.K., Li J.H., Lei J.J., Xu Q.H., Ma J.G., Li X.Q. (2017). Desmoplasia suppression by metformin-mediated AMPK activation inhibits pancreatic cancer progression. Cancer Lett..

[50] Lamouille S., Connolly E., Smyth J.W., Akhurst R.J., Derynck R. (2012). TGf-β-induced activation of mTOR complex 2 drives epithelial-mesenchymal transition and cell invasion. Development.

[51] Khezri M.R., Melekinejad H., Majidi-Zolbanin N., Ghasemnejad-Berenji M. (2021). Anticancer potential of metformin: Focusing on gastrointestinal cancers. Cancer Chemother. Pharmacol..

[52] Ma R., Yi B., Riker A.I., Xi Y. (2020). Metformin and cancer immunity. Acta Pharmacol. Sin..

[53] Gyawali M., Venkatesan N., Ogeyingbo O.D., Bhandari R., Botleroo R.A., Kareem R., Ahmed R., Elshaikh A.O. (2021). Magic of a Common Sugar Pill in Cancer: Can Metformin Raise Survival in Pancreatic Cancer Patients?. Cureus.

[54] De Souza A., Khawaja K.I., Masud F., Saif M.W. (2016). Metformin and pancreatic cancer: Is there a role?. Cancer Chemother. Pharmacol..

[55] Duan W.X., Qian W.K., Zhou C.C., Cao J.Y., Qin T., Xiao Y., Cheng L., Li J., Chen K., Li X.Q. (2018). Metformin suppresses the invasive ability of pancreatic cancer cells by blocking autocrine TGF-ß1 signaling. Oncol. Rep..

[56] Wu Q., Miele L. (2012). The Role of EMT in Pancreatic Cancer Progression. Pancreat. Disord. Ther..

[57] Incio J., Suboj P., Chin S.M., Vardam-Kaur T., Liu H., Hato T., Babykutty S., Chen I., Deshpande V., Jain R.K. (2015). Metformin reduces desmoplasia in pancreatic cancer by reprogramming stellate cells and tumor-associated macrophages. PLoS ONE.

[58] Zi F., Zi H., Li Y., He J., Shi Q., Cai Z. (2018). Metformin and cancer: An existing drug for cancer prevention and therapy (review). Oncol. Lett..

[59] Piffoux M., Eriau E., Cassier P.A. (2021). Autophagy as a therapeutic target in pancreatic cancer. Br. J. Cancer.

[60] Galluzzi L., Pietrocola F., Bravo-San Pedro J.M., Amaravadi R.K., Baehrecke E.H., Cecconi F., Codogno P., Debnath J., Gewirtz D.A., Karantza V. (2015). Autophagy in malignant transformation and cancer progression. EMBO J..

[61] Morales D.R., Morris A.D. (2015). Metformin in cancer treatment and prevention. Annu. Rev. Med..

[62] New M., Van Acker T., Long J.S., Sakamaki J.I., Ryan K.M., Tooze S.A. (2017). Molecular pathways controlling autophagy in pancreatic cancer. Front. Oncol..

[63] Iliopoulos D., Hirsch H.A., Struhl K. (2011). Metformin decreases the dose of chemotherapy for prolonging tumor remission in mouse xenografts involving multiple cancer cell types. Cancer Res..

[64] Yu X., Mao W., Zhai Y., Tong C., Liu M., Ma L., Yu X.L., Li S.S. (2017). Anti-tumor activity of metformin: From metabolic and epigenetic perspectives. Oncotarget.

[65] Lonardo E., Cioffi M., Sancho P., Sanchez-Ripoll Y., Trabulo S.M., Dorado J., Balic A., Hidalgo M., Heeschen C. (2013). Metformin Targets the Metabolic Achilles Heel of Human Pancreatic Cancer Stem Cells. PLoS ONE.

[66] Xin W., Fang L., Fang Q., Zheng X., Huang P. (2017). Effects of metformin on survival outcomes of pancreatic cancer patients with diabetes: A meta-analysis. Mol. Clin. Oncol..

[67] Saini N., Yang X. (2018). Metformin as an anti-cancer agent: Actions and mechanisms targeting cancer stem cells. Acta Biochim. Biophys. Sin..

[68] Sahra I.B., Regazzetti C., Robert G., Laurent K., Le Marchand-Brustel Y., Auberger P., Tanti J.F., Giorgetti-Peraldi S., Bost F. (2011). Metformin, independent of AMPK, induces mTOR inhibition and cell-cycle arrest through REDD1. Cancer Res..

[69] Hassan Z., Schneeweis C., Wirth M., Veltkamp C., Dantes Z., Feuerecker B., Ceyhan G.O., Knauer S.K., Weichert C., Schmid R.M. (2018). MTOR inhibitor-based combination therapies for pancreatic cancer. Br. J. Cancer.

[70] Papaconstantinou I.G., Manta A., Gazouli M., Lyberopoulou A., Lykoudis P.M., Polymeneas G., Voros D. (2013). Expression of MicroRNAs in Patients With Pancreatic Cancer and Its Prognostic Significance. Pancreas.

[71] Funamizu N., Ray Lacy C., Kamada M., Yanaga K., Manome Y. (2019). MicroRNA-200b and -301 are associated with gemcitabine response as biomarkers in pancreatic carcinoma cells. Int. J. Oncol..

[72] Jafri M.A., Al-Qahtani M.H., Shay J.W. (2017). Role of miRNAs in human cancer metastasis: Implications for therapeutic intervention. Semin. Cancer Biol..

[73] Zhang W.L., Zhang J.H., Wu X.Z., Yan T., Lv W. (2015). MiR-15b promotes epithelial-mesenchymal transition by inhibiting SMURF2 in pancreatic cancer. Int. J. Oncol..

[74] Bai Z., Sun J., Wang X., Wang H., Pei H., Zhang Z. (2015). MicroRNA-153 is a prognostic marker and inhibits cell migration and invasion by targeting SNAI1 in human pancreatic ductal adenocarcinoma. Oncol. Rep..

[75] Liu G., Ji L., Ke M., Ou Z., Tang N., Li Y. (2018). miR-125a-3p is responsible for chemosensitivity in PDAC by inhibiting epithelial-mesenchymal transition via Fyn. Biomed. Pharmacother..

[76] Hiramoto H., Muramatsu T., Ichikawa D., Tanimoto K., Yasukawa S., Otsuji E., Inazawa J. (2017). MiR-509-5p and miR-1243 increase the sensitivity to gemcitabine by inhibiting epithelial-mesenchymal transition in pancreatic cancer. Sci. Rep..

[77] Pan G., Liu Y., Shang L., Zhou F., Yang S. (2021). EMT-associated microRNAs and their roles in cancer stemness and drug resistance. Cancer Commun..

[78] Cufí S., Vazquez-Martin A., Oliveras-Ferraros C., Quirantes R., Segura-Carretero A., Micol V., Joven J., Bosch-Barrera J., Del Barco S., Martin-Castillo B. (2012). Metformin lowers the threshold for stress-induced senescence: A role for the microRNA-200 family and miR-205. Cell Cycle.

[79] Bao B., Wang Z., Ali S., Ahmad A., Azmi A.S., Sarkar S.H., Banerjee S., Kong D., Li Y.W., Thakur S. (2012). Metformin inhibits cell proliferation, migration and invasion by attenuating CSC function mediated by deregulating miRNAs in pancreatic cancer cells. Cancer Prev. Res..

[80] Ma M.L., Ma C.F., Li P.P., Ma C.X., Ping F., Li W., Xu L.L., Zhang H.B., Sun Q., Li Y.X. (2020). Low glucose enhanced metformin’s inhibitory effect on pancreatic cancer cells by suppressing glycolysis and inducing energy stress via up-regulation of miR-210-5p. Cell Cycle.

[81] Nimmakayala R.K., Leon F., Rachagani S., Rauth S., Nallasamy P., Marimuthu S., Shailendra G.K., Chhonker Y.S., Chugh S., Chirravuri R. (2021). Metabolic programming of distinct cancer stem cells promotes metastasis of pancreatic ductal adenocarcinoma. Oncogene.

[82] Andriani F., Bertolini G., Facchinetti F., Baldoli E., Moro M., Casalini P., Caserini R., Milione M., Leone G., Pelosi G. (2016). Conversion to stem-cell state in response to microenvironmental cues is regulated by balance between epithelial and mesenchymal features in lung cancer cells. Mol. Oncol..

[83] Luo M., Wicha M.S. (2015). Metabolic plasticity of cancer stem cells. Oncotarget.

[84] Zhang H.H., Guo X.L. (2016). Combinational strategies of metformin and chemotherapy in cancers. Cancer Chemother. Pharmacol..

[85] Lonardo E., Cioffi M., Sancho P., Crusz S., Heeschen C. (2015). Studying Pancreatic Cancer Stem Cell Characteristics for Developing New Treatment Strategies. J. Vis. Exp..

[86] Sancho P., Barneda D., Heeschen C. (2016). Hallmarks of cancer stem cell metabolism. Br. J. Cancer.

[87] Suzuki K., Takeuchi O., Suzuki Y., Kitagawa Y. (2019). Mechanisms of metformin’s anti-tumor activity against gemcitabine-resistant pancreatic adenocarcinoma. Int. J. Oncol..

[88] Kordes S., Pollak M.N., Zwinderman A.H., Mathôt R.A., Weterman M.J., Beeker A., Punt C.J., Richel D.J., Wilmink J.W. (2015). Metformin in patients with advanced pancreatic cancer: A double-blind, randomised, placebo-controlled phase 2 trial. Lancet Oncol..

[89] Broadhurst P.J., Hart A.R. (2018). Metformin as an Adjunctive Therapy for Pancreatic Cancer: A Review of the Literature on Its Potential Therapeutic Use. Dig. Dis. Sci..

[90] Reni M., Dugnani E., Cereda S., Belli C., Balzano G., Nicoletti R., Liberati D., Pasquale V., Scavini M., Maggiora P. (2016). (Ir)relevance of Metformin Treatment in Patients with Metastatic Pancreatic Cancer: An Open-Label, Randomized Phase II Trial. Clin. Cancer Res..

[91] Rena G., Hardie D.G., Pearson E.R. (2017). The mechanisms of action of metformin. Diabetologia.

[92] Elgogary A., Xu Q., Poore B., Alt J., Zimmermann S.C., Zhao L., Fu J., Chen B.W., Xia S.Y., Liu Y.F. (2016). Combination therapy with BPTES nanoparticles and metformin targets the metabolic heterogeneity of pancreatic cancer. Proc. Natl. Acad. Sci. USA.

[93] Wei M., Liu Y., Bi Y., Zhang Z.-J. (2019). Metformin and pancreatic cancer survival: Real effect or immortal time bias?. Int. J. Cancer.

[94] Frouws M.A., Mulder B.G.S., Bastiaannet E., Zanders M.M.J., Van Herk-Sukel M.P.P., De Leede E.M., Bonsing B.A., Mieog J.S., Van de Velde C.J.H., Liefers G.-J. (2017). No association between metformin use and survival in patients with pancreatic cancer. Medicine.

[95] Dong Y.-W., Shi Y.-Q., He L.-W., Cui X.-Y., Su P.-Z. (2017). Effects of metformin on survival outcomes of pancreatic cancer: A meta-analysis. Oncotarget.

[96] Singh R.R., O’reilly E.M. (2020). New Treatment Strategies for Metastatic Pancreatic Ductal Adenocarcinoma. Drugs.

